# Numerical study of perforated obstacles effects on the performance of solar parabolic trough collector

**DOI:** 10.3389/fchem.2022.1089080

**Published:** 2023-01-17

**Authors:** Tayeb Fahim, Samir Laouedj, Aissa Abderrahmane, Zied Driss, El Sayed Mohamed Tag-ElDin, Kamel Guedri, Obai Younis

**Affiliations:** ^1^ Materials and Reactive Systems Laboratory (LMSR), Djillali Liabes University, Sidi Bel Abbes, Algeria; ^2^ Laboratoire de Physique Quantique de la Matière et Modélisation Mathématique (LPQ3M), Université Mustapha Stambouli de Mascara, Mascara, Algeria; ^3^ Laboratory of Electromechanical Systems (LASEM), National School of Engineers of Sfax, University of Sfax, Sfax, Tunisia; ^4^ Center of Research, Faculty of Engineering, Future University in Egypt, New Cairo, Egypt; ^5^ Mechanical Engineering Department, College of Engineering and Islamic Architecture, Umm Al-Qura University, Makkah, Saudi Arabia; ^6^ Department of Mechanical Engineering, College of Engineering in Wadi Addwasir, Prince Sattam Bin Abdulaziz University, Al-kharj, Saudi Arabia

**Keywords:** nanofluid, parabolic trough solar collector, Nusselt number, perforated obstacles, numerical investigation

## Abstract

The current work presents and discusses a numerical analysis of improving heat transmission in the receiver of a parabolic trough solar collector by introducing perforated barriers. While the proposed approach to enhance the collector’s performance is promising, the use of obstacles results in increased pressure loss. The Computational Fluid Dynamics (CFD) model analysis is conducted based on the renormalization-group (RNG) k-ɛ turbulent model associated with standard wall function using thermal oil D12 as working fluid The thermo-hydraulic analysis of the receiver tube with perforated obstacles is taken for various configurations and Reynolds number ranging from 18,860 to 81,728. The results are compared with that of the receiver without perforated obstacles. The receiver tube with three holes (PO3) showed better heat transfer characteristics. In addition, the Nusselt number (Nu) increases about 115% with the increase of friction factor 5–6.5 times and the performance evaluation criteria (PEC) changes from 1.22 to 1.24. The temperature of thermal oil fluid attains its maximum value at the exit, and higher temperatures (462.1 K) are found in the absorber tube with perforated obstacles with three holes (PO3). Accordingly, using perforated obstacles receiver for parabolic trough concentrator is highly recommended where significant enhancement of system’s performance is achieved.

## Highlights

- The flow and thermal characteristics of through solar collector was examined.
- The benefits effects of using perforated baffles to enhance heat transfer was analyzed.
- The position and number of perforations was optimized to obtain the best heat transfer.

## Introduction

Growth in global energy demand and the overuse of non-renewable energy sources such as petrol and natural gas have reduced these resources’ availability and resulted in harmful severe environmental consequences such as air pollution and global warming (Jamshed et al., 2021; Wu et al., 2021; Zandalinas et al., 2021). Researchers focused on improving technologies involved in renewable energy sources such as solar to address these issues. Solar collectorsuse a heat-exchanging fluid to convert solar power to thermal power. In fact, using the absorber tube absorbs solar light and transfers heat to the absorber fluid. Therefore, the solar collector increases its internal energy, which may be utilized for other purposes (Sayed et al., 2020; Pandey et al., 2021; Shahzad et al., 2021).

Changing traditional working fluids in solar collectors to nanofluids is one of the activities that has gotten a lot of attention in recent years to improve their thermal performance (Aman et al., 2015; Fares et al., 2020; Mourad et al., 2021; Hassan et al., 2022; Khalid et al., 2022). Dehaj et al. (2021) designed and developed an experimental test bench to examine the parabolic trough solar collector (PTSC) efficiency using NiFe_2_O_4_/Water nanofluid as a working fluid. They used a U-shaped absorber tube. Their results show that the PTSC was more effective when the Nickel Ferrite nanofiuid was introduced. In fact, for a volumetric fraction of .05% and a fiow rate of 3 L/min, an efficiency of 51% can be achieved. Farhana et al. (Farhana et al., 2021)investigated the flat plate solar collector efficiency with crystal nano-cellulose (CNC) nanofluid through an experiment. They revealed that the efficiency of the FPSC was enhanced by 2.48% and 8.46% when .5% Al_2_O_3_ and .5% CNC nanofluids were used, respectively.

Hosseini et al. (Hosseini Seyed and Shafiey Dehaj, 2021) calculated the energetic performance of a PTSC working with Al_2_O_3_ and GO nanofiuid with a .2% volume fraction. They found that the thermal efficiency of the PTSC was improved by 63.2% and 32.1% when the GO nanofiuid and the Al_2_O_3_ nanofiuid were used, respectively. Vahidinia et al. (Vahidinia et al., 2021) valued the thermal performance of the PTSC using three types of Syltherm 800 based nanofluids. The first two are Al_2_O_3_ and SiO_2,_ and the third is a hybrid nanofluid merging the above two. They illustrated that the exergy and energy performance of the hybrid nanofluid is always the highest. Vital et al. (Vital Caio. et al., 2021) evaluated the thermo-optical properties of TMN nanofluids such as TiN, ZrN, and HfN in an aqueous medium where the nanofluids were used as working fluids for a direct absorption solar collector (DASC). According to their results, the efficiencies of DASC employing TiN, ZrN, and HfN NF are 6.3%, 5.2%, and 5.6%, respectively. They also stated that these enhancements could be achieved without increasing the demands of pumping power by using a low-concentration regime. Ould-Lahoucine et al. (Ould-Lahoucine et al., 2021) proposed a novel technique to identify the ideal height of the rectangular cooling channel for photovoltaic/thermal (PV/T) collector system employing TiO_2_-water nanofluid. Before that, they discussed this nanofluid’s energy and exergy performances inside the PV/T.

Some researchers focused on nanofluid flow through the absorber tube, which is essentially a channel. Esmaeili et al. (Esmaeili et al., 2019) applied a two-phase model to inspect turbulent flow with both forced and free convection of nanofluid within a 3D rectangular channel (Ajeel Raheem et al, 2022). numerically analyzedthe flow pattern and heat transfer properties of ZnO-water nanofluid within a new channel, where both curved and corrugated profiles for the walls and E-shaped baffles. Berrehal et al. (Berrehal and Sowmya, 2021) analyzed nanofluid flow between two inclined walls using the optimal homotopy asymptotic technique. Ajeel et al. (Ajeel Raheem. et al., 2021) utilized the multi-phase mixture model to evaluate the thermal-hydraulic performance of binary hybrid nanofiuid flowing within a curved-corrugated channel. The results show that using the binary hybrid nanoparticles enhanced the thermal characteristics of the base fluid, thus improving the heat transfer rate in the system. This effect can be furthered by raising the volume fraction or the blockage ratio and reducing the pitch angle.

Recently, a new technique has been employed to enhance nanofluid’s heat transport and flow inside channels. It consists of inserting a vortex generator of various shapes and sizes. Their primary purpose is to increase the flow turbulence intensity and eliminate the laminar boundary layer near the walls of the channels. Maadi et al. (Seyed Reza et al., 2021) attempted to enhance the performance of a photovoltaic-thermal system (PV/T) by employing nanofiuid and a wavy-strip insert. The outcomes show that using Al_2_O_3_water-based nanofiuid and wavy-strip inserts improved the PVT system’s thermal efficiency by 12.06% compared to typical PVT. Mashayekhi et al. (Ramin et al., 2020) analyzed the impact of two rows of twisted conical strip inserts on the flow of a water-Al_2_O_3_ nanofluid in an oval tube. Their study illustrated that inward Co-Conical inserts provide the highest value of heat transport rate, as it can reach 17% higher than tube without inserts. Hamid et al. (Hamid et al., 2019) performed experiments to study the combined impacts of using TiO_2_–SiO_2_ nanofluids and wire coil inserts on a tube’s flow and heat transfer. Chadi et al. (Kamel et al., 2021) studied a diamond-water nanofluid’s heat transfer and flow through micro-channels fitted with parallelogram ribs and pie-shaped ribs. The outcomes show that the heat transfer rate was highest when the parallelogram ribs were used. Jing et al. (Jing et al., 2020) underlined the significance of the magnetic field and the shape of heating fins on the flow and heat transport in a rectangular enclosure loaded with nanofluid. Azmi et al. (Azmi et al., 2021) scrutinized the performance of TiO_2_–SiO_2_/water hybrid nanofluid with various composition ratios flowing inside a tube equipped with wire coil inserts. The outcomes showed that the highest thermal performance factor reached (1.72) with a composition ratio R = .2. In addition, the wire coils can enhance the heat transfer of TiO_2_–SiO_2_nanofluids by up to 211.75%. Rathnakumar et al. (Rathnakumar et al., 2014) considered improving heat transport turbulent fiow in a tube by equipping it with helical screw louvered rod inserts and employing (CNT)/water nanofiuids at various volume concentrations. The calculations indicated that the helical louvered rod inserts cause augmentation in heat transfer for a certain Reynolds number compared to a plain tube, whereas the friction factor also increased. Kumar et al. (Kumar et al., 2018) and Sundar et al. (Syam et al., 2020) explored the effect of twisted tape and wire coil with core-rod inserts on the heat transport, the friction factor of Fe_3_O_4_/water nanofiuid fiow inside a double pipe U-bend heat exchanger. Sundar et al. (Syam, Said, Saleh, Singh, Antonio Sousa) calculated the thermal-hydraulic performance of rGO/Co_3_O_4_ hybrid nanofluid in a plain horizontal tube and another one fitted with longitudinal strip inserts. Their findings indicate that the Nusselt number is boosted by 25.65% when the concentration of hybrid nanoparticles in water is .2%. It is further improved by 110.56% when a straight strip is inserted. However, employing linear strip inserts and hybrid nanofluids results in aninsignificantdrawback in fluid friction. Alnaqi et al. (Abdulwahab et al., 2021)examined the performance of a solar collector fitted with two twisted tape inserts and loaded with MgO-MWCNT thermal oil-based hybrid nanofluid. Mohammed et al. (Hussein et al., 2019) studied numerically the overall thermo-hydraulic performance of nanofiuids in forced convection fiow inside circular tubes fitted with divergent and convergent conical rings inserts. According to their results, the divergent ring inserts produced a 365% enhancement in the performance criteria, making them the best option. Sheikhzadeh et al. (Ghanbar et al., 2019) examined an ethylene-glycol-based hybrid nanofluid’s thermodynamic and flow properties in a rectangular channel with turbulators with various wing forms. The result shows that the trapezoidal wings with a volume fraction of .6% provide the best heat transfer performance considering fluid flow.

In recent years, various combinations of nanofluids as well as affecting parameters on the different structures are taken for analysis-oriented with the thermal application such as solar collectors. They have been considered and developed, as a result, effective enhancement of heat transfer achieved by many research works (Rathnakumar et al., 2014; Kumar et al., 2018; Ghanbar et al., 2019; Hamid et al., 2019; Hussein et al., 2019; Jing et al., 2020; Ramin et al., 2020; Syam et al., 2020; Syam, Said, Saleh, Singh, Antonio Sousa; Abdulwahab et al., 2021; Azmi et al., 2021; Kamel et al., 2021; Seyed Reza et al., 2021). To the authors’ knowledge, no research has been done on the examination of the position and number of perforations to optimize and to enhance the performance of a photovoltaic-thermal system (PV/T) by employing nanofiuid and a wavy-strip insert. Accordingly, the aim of this study is to improve heat transfer inside parabolic through solar receiver using two different passive methods, perforated obstacles and nanoparticles. This work also investigates the effects of perforated obstacles inserted centrally inside the absorber of a PTC for various cases. Inserts are placed centrally, the diameters of the perforations are large, they are fixed to the top surface of the receiver tube, and their dimensions are much smaller than those found in the literature. In reality, inserting holes in obstacles aids fluid mixing by breaking the thermal boundary layer and aids fluid mixing due to the non-uniform circumferential heat flow profile on the receiver; hence the thermal performance is remarkably improved. The motivated work aims to answer to the following research questions.

- What is the effect of the perforated obstacles on heat transfer in a solar parabolic trough collector using nanofluids?
- What is the impact of the position and number of these perforations on the temperature of thermal oil fluid used?
- What are the benefits effects of using perforated baffles to enhance heat the performance evaluation criteria.
- How friction factor values decrease with the increase in the number of these holes?

## Model description

### Physical model

**FIGURE 1:**
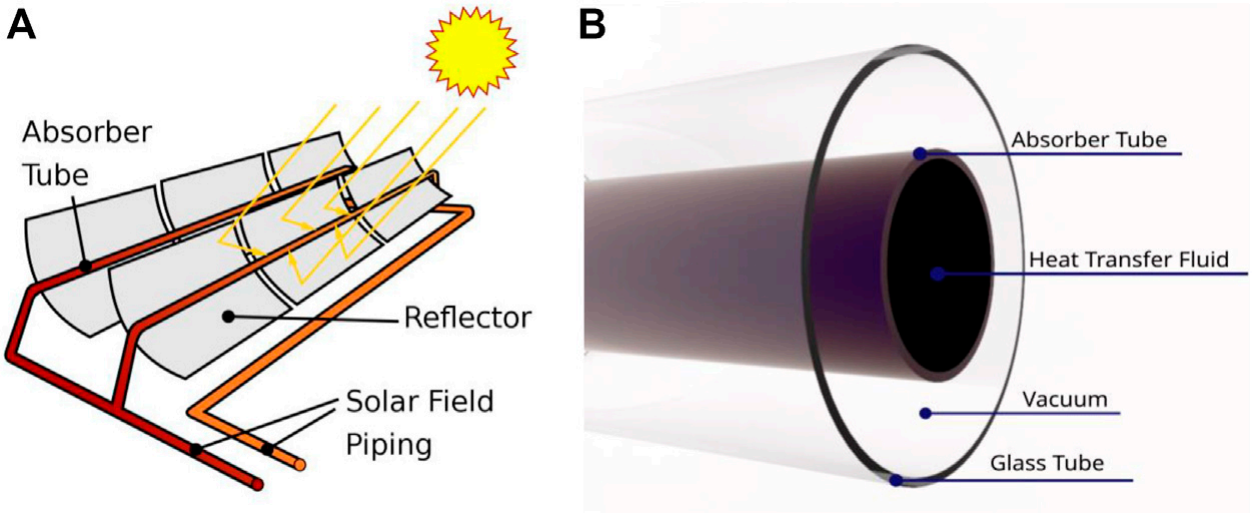
**(A)** Parabolic trough collector (PTC). **(B)** Cross-sectional diagram of the parabolic trough receiver (PTR) (Wang et al., 2015).

**FIGURE 2:**
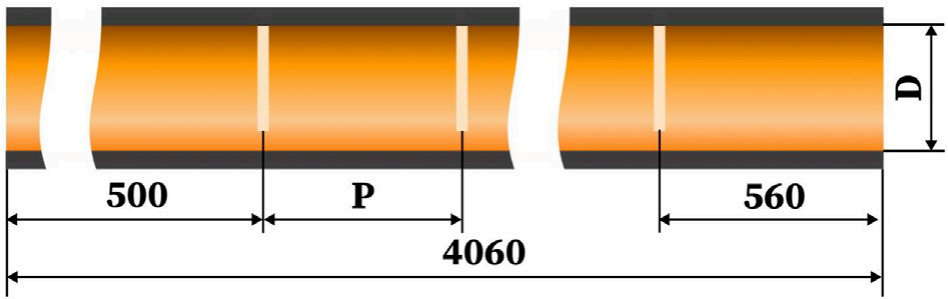
The receiver’s longitudinal segment with perforated obstruction inserts.

**FIGURE 3:**
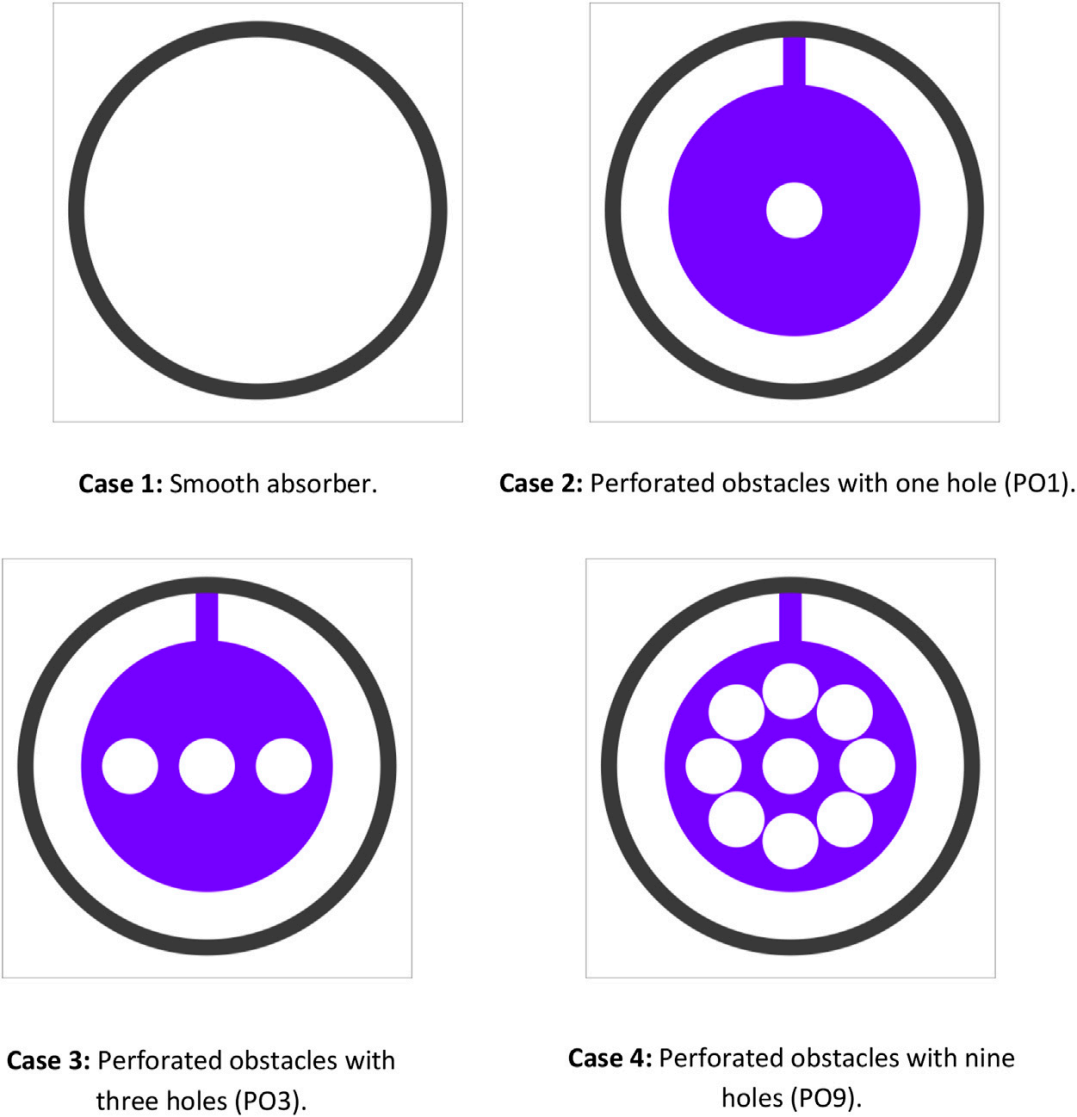
Cross-section of the receiver tube with perforated obstacles and smooth absorber.

Figure 1 depicts the solar parabolic system’s schematic and the collector’s receiver. The parabolic trough collector (PTC) concentrates the direct sunlight on the bottom perimeter of the parabolic trough receiver (PTR). In contrast, the top perimeter of the PTR is exposed to non-concentrated solar irradiation (Figure 1A). Figure 1 shows a cross-section drawing of the PTR (b). A glass cover protects the stainless-steel absorber tube. The space between the metal tube and the glass cover is constantly vacuumed to reduce heat loss. The focused solar energy travels through the glass cover and lands on the metal tube’s outer surface. The concentrated solar energy is absorbed and transformed into heat by the metal tube. The heat is conveyed to the heat transfer fluid by conduction and convection modes. The receiver model employed in this study intends to improve the heat transmission performance of PTR by introducing absorber tubes with perforated barriers (Figures 2, 3). Table 1 shows the geometrical characteristics of PTR and perforated barriers. Table 2 also depicts the thermophysical parameters of the working fluid (Thermal oil D12), perforated barriers, and absorber tube.

**TABLE 1:** The PTR model parameters and perforated obstacles.

Parameter	Values	Reference
Length of the absorber (m)	4.06	Wu et al., 2014a, Wu et al., 2014b
Internal diameter of the metal tube (m)	.064	
External diameter of the metal tube (m)	.07	
Internal diameter of the Glass cover (m)	.117	
External diameter of the Glass cover (m)	.12	
Glass envelope transmissivity	.95	
Metal tube absorptivity	.96	
Obstacle diameter (mm)	46	
Perforated obstaclesthickness (mm)	2	
Diameter of the perforation (mm)	10	
Distance between two consecutive perforated obstacles (mm)	128	
Number of perforated obstacles in the absorber tube	25	

**TABLE 2:** Thermophysical properties of the working fluid, perforated obstacles, and absorber tube.

	Working fluid (thermal oil D12)	Perforated obstacles and absorber tube (stainless steel)	Reference
Density (Kg/m3)	679	8,027	Solutia, (1998)
Specific heat (J/Kg.K)	2,571	500	
Thermal conductivity (W/m.K)	.091	20	
Viscosity (N.s/m2)	.000346	—	

### Boundary conditions

The boundary conditions are as follows.

• Fluid inlet:

$$V_{x=}V_{in},\;V_{y=}V_{z}=0m/s,$$ (1)

$$T_{f}=T_{\mathrm{in}}=400\;K\;(L=0,\;00^{0}\leq \varphi \leq 360{}^{\circ}$$ (2)

• At the walls

The upper half perimeter of the metal tube is exposed to the uniform heat flux qt, which is calculated as:

$$q_{t}=DNI\times TGE\times AMT=1000\times 0.95\times 0.96=912W/m^{2}\left(0\leq L\leq 4.06m,0⁰\leq \varphi \leq 180⁰\right)$$ (3)

**FIGURE 4:**
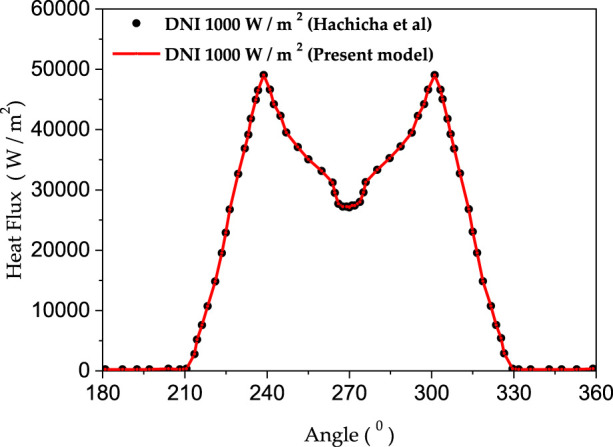
Heat flux distribution variation along the bottom half perimeter of the absorber tube.

Where DNI, TGE, and AMT are the solar irradiation, the glass envelope transmissivity, and the metal tube absorptivity, respectively. The concentrated solar irradiation qcal was computed by. (Kamel et al., 2021) (Figure 4). The lower half perimeter of the metal tube is subjected to the heat flux qb, which is calculated as:

$$q_{b}=q_{cal}\;;\;\left(0\leq L\leq 4.06\;m,\;180{}^{\circ}\leq \varphi \leq 360{}^{\circ}\right)$$ (4)

**FIGURE 5:**
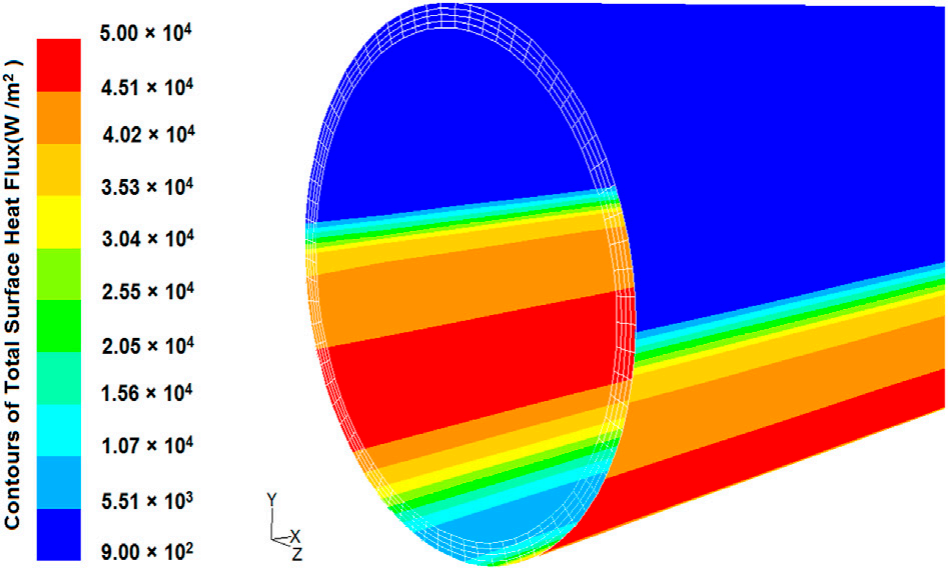
Heat flux distribution on the absorber tube surface (current study).

• Fully formed conditions are enforced at the fluid outflow.
• In this study, the outer absorber wall is subjected to a non-uniform heat flux estimated using the Monte Carlo Ray Tracing (MCR) method and a DNI of 1000 W/m2. Figure 4 depicts the variation of the heat flux distribution along the bottom-half perimeter of the absorber tube for present and Hachicha et al. (Hachicha et al., 2013) models. Using the current calculation, the heat flux distribution pattern of the absorber tube is plotted in Figure 5.

## Numerical model

### Numerical method

**FIGURE 6:**
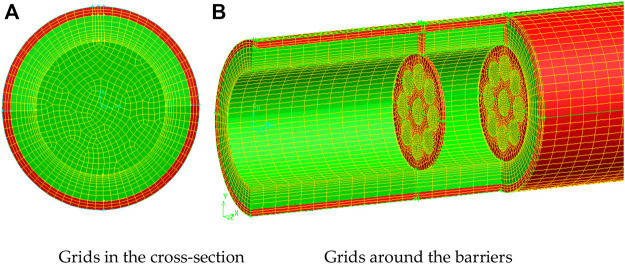
3D mesh of the computational domain.

The computational fluid dynamics (CFD) modeling technique was used in this work. The finite volume technique (FVM) is used to discretize the equations. The resulted equations system is solved numerically by employing the commercial package software ANSYS-FLUENT (Release 17.1). The RNG k-ɛ turbulence model is employed to simulate the turbulent flow of Thermal oil D12 in the solar collector’s absorber tube. Second-order UPWIND and QUICK methods are used for discretizing the convective components in momentum and energy equations. For dealing with pressure-velocity coupling, the SIMPLEC method is utilized. For all equations, the convergence threshold is 10–6. GAMBIT version 2.2 is used to generate and the mesh of the physical model (Figure 6).

### Governing equations

The instantaneous Navier Stokes equation is used to generate the RNG k-turbulent model by using a mathematical approach known as “renormalization group” (RNG) methods (Gnielinski, 1976; Yakhot et al., 1992). The values of k (turbulent kinetic energy) and ɛ (turbulent dissipation rate) are determined by equations:

$$\rho \left(\frac{\partial k}{\partial t}+\bar{u_{j}}\frac{\partial k}{\partial x_{j}}\right)=\frac{\partial }{\partial x_{j}}\left[\left(\mu +\frac{\mu_{t}}{\sigma_{k\;\left(RNG\right)}}\right)\frac{\partial k}{\partial x_{j}}\right]+P_{k}-\rho \varepsilon$$ (5)

$$\rho \left(\frac{\partial \varepsilon }{\partial t}+\bar{u_{i}}\frac{\partial \varepsilon }{\partial x_{i}}\right)=\frac{\partial }{\partial x_{j}}\left[\left(\mu +\frac{\mu_{t}}{\sigma_{\varepsilon \;\left(RNG\right)}}\right)\frac{\partial \varepsilon }{\partial x_{j}}\right]+\frac{\varepsilon }{k}\left(C_{1\varepsilon \left(RNG\right)}P_{k}-C_{2\varepsilon \left(RNG\right)}\rho \frac{\varepsilon }{k}\right)$$ (6)

Where

$$P_{k}=-\rho \bar{{u_{i}}^{\prime }{u_{j}}^{\prime }}\frac{\partial \bar{u_{i}}}{\partial x_{j}}$$ (7)

$$C_{2\varepsilon \left(RNG\right)}=\frac{C_{2\varepsilon }+C_{\mu }\eta^{3}\left(1-\frac{\eta }{\eta_{0}}\right)}{1+\beta \eta^{3}}$$ (8)

$$\eta =\frac{k}{\varepsilon }{\left(2S_{ij}S_{ij}\right)}^{1/2}$$ (9)

The turbulent viscosity $\mu_{t}$ is calcluated as:

$$\mu_{t}=\rho C_{\mu }\frac{k^{2}}{\varepsilon }$$ (10)

Where the parameter ρ represents the fluid’s density.

$S_{ij}$ denotes the strain tensor rate and is defined as:

$$S_{ij}=\frac{1}{2}\left(\frac{\partial \bar{u_{i}}}{\partial x_{j}}+\frac{\partial \bar{u_{j}}}{\partial x_{i}}\right)$$ (11)

Table 3 summarizes the model constants used.

**TABLE 3:** Model constants.

$C_{\mu }$	$C_{1\varepsilon \left(RNG\right)}$	$C_{2\varepsilon \left(RNG\right)}$	$\sigma_{k\;\left(RNG\right)}$	$\sigma_{\varepsilon \;\left(RNG\right)}$	$\eta_{0}$	β
.0845	1.42	1.68	.7194	.7194	4.38	.012

## Results and discussion

### Grid independency

Numerous calculations were undertaken to determine the total number of grid points required to create an array adequate for measuring flux and thermal field in order to justify the simulation solution’s accuracy and consistency. Table 4 illustrates the evolution of the average Nusselt number as a cell number function for Reynolds numbers ranging from 10^4^ to 10^6^.

**TABLE 4:** Mesh effect on the average Nusselt number.

N_cells_					
	294600	307200	330400	354400	
**Re**	Nu				|δmax|
10^4^	148.123	150.021	149.613	150.461	1.57%
10^5^	222.104	226.371	226.719	229.004	3.10%
10^6^	259.121	258.223	259.942	260.781	.64%

### Code validation

To determine the validity and correctness of the model and numerical solution used in this inquiry, the Nusselt number generated in this study is compared to the Nusselt number computed using the Gnielinski correlation (Petukhov et al., 1970). Gnielinski devised the following equation to get the Nusselt number of a smooth tube:

$${Nu}_{D}=\frac{\left(f/8\right)\left(R_{eD}-1000\right)\mathrm{Pr}}{1+12.7{\left(f/8\right)}^{1/2}\left({\mathrm{Pr}}^{2/3}-1\right)}\;;\;For\;3000\leq Re\leq 5\times 10^{6}and\;0.5\leq \mathrm{Pr}\leq 2000$$ (12)

Where the Petukhov friction correlation is as used in (Gee and Webb, 1980):

$$f={\left(0.790\;\ln{Re}_{D}-1.64\right)}^{-2}\;;\;For\;3000\leq Re\leq 5\times 10^{6}$$ (13)

By flowing the PTR heat transfer fluid through a metal tube, the heat transfer properties of the fluid are studied. The Nu_avg_, Re, and heat transfer coefficient (h) read are as follows:

$${Nu}_{avg}=\frac{h.D}{\lambda }$$ (14)

$$Re=\frac{D.v}{\nu }$$ (15)

$$h=\frac{q^{\prime \prime }}{T_{t,a}-T_{f,a}}$$ (16)

The Darcy friction factor in turbulent flow regime is as defined in (Amina et al., 2017):

$$f=\frac{2.∆P.D}{L.\rho .v^{2}}$$ (17)

Using the relation between the pressure and shear forces, the above expression can be written as:

$$f=\frac{8.\tau_{w}}{\rho .v^{2}}$$ (18)

**FIGURE 7:**
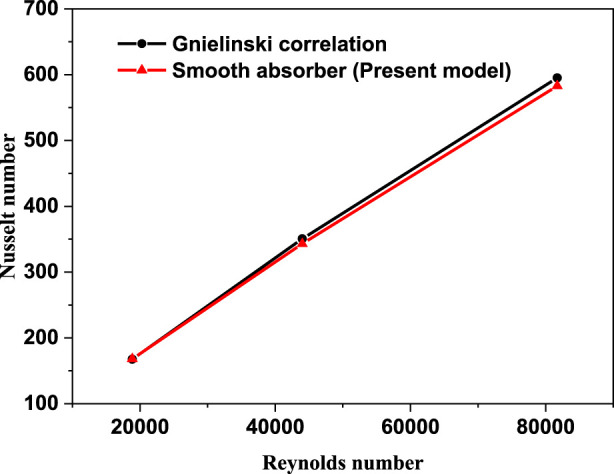
Nussle number with Gnielinski correlation.

**FIGURE 8:**
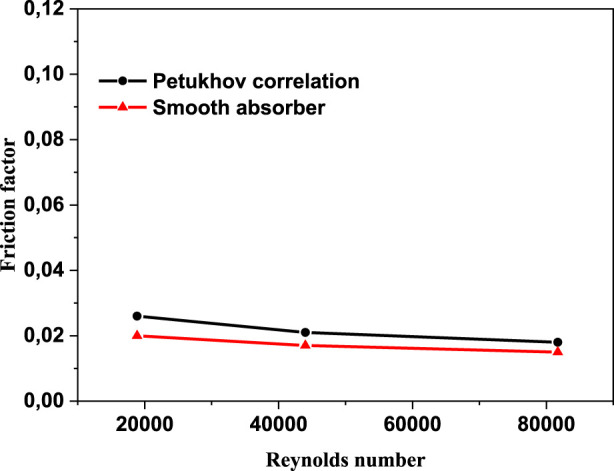
Friction factor with Petukhov correlation.

To verify the quality of the computational model employed in this study, the Gnielinski and Petukhov correlations for the Nusselt number and friction factor are utilized to evaluate the simulation of heat transfer and flow properties of the thermal oil D12 in the absorber tube. Figure 7 and Figure 8 show the friction factor and Nusselt number comparisons between the numerical results and the correlations for smooth absorber, respectively. The maximum deviation value of the numerical results was found to be around 7.8% and 15%, and the minimum deviation equals 18% and 11% for the Nusselt number and friction factor, respectively. The heat transfer and flow properties are clearly in agreement with the correlations.

### Effect of perforated obstacles on heat transfer

**FIGURE 9:**
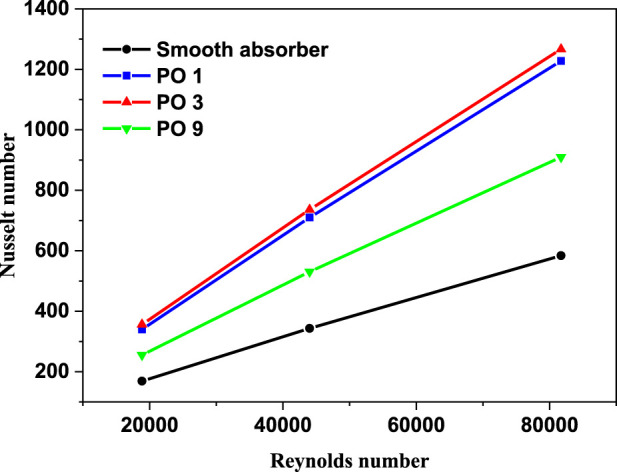
Nu variation in the absorber vs. Reynolds number (P/D = 2).

As seen in Figure 9, changes in Reynolds number (Re) generate fluctuations in Nusselt number, which has values of 18860 (v = 15 m/s), 44007 (v = 35 m/s), and 81728 (v = 65 m/s) when using thermal oil D12 as working fluid. The Nusselt number approximately linearly rises in proportion to the Reynolds number; this enhancement is caused by introducing perforated barriers, which improve the heat transfer area. The vortex flow was caused by fluid mixing given by the perforated barriers, and enhanced turbulent intensity at high values of Re leads the thermal boundary layer to be destroyed. The highest gain is seen in absorber tubes with three holes and perforated barriers (PO3). The average Nusselt number improves by 115 percent compared to the standard case with the smooth absorber. Perforated barriers with one hole (PO1) are the second most successful example, with an average Nusselt number enhancement of 108 percent.

**FIGURE 10:**
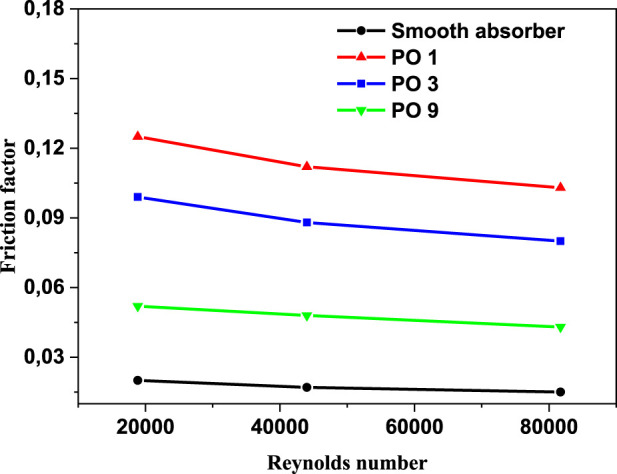
Variation in absorber tube’s friction factor vs. Reynolds number (P/D = 2).

**FIGURE 11:**
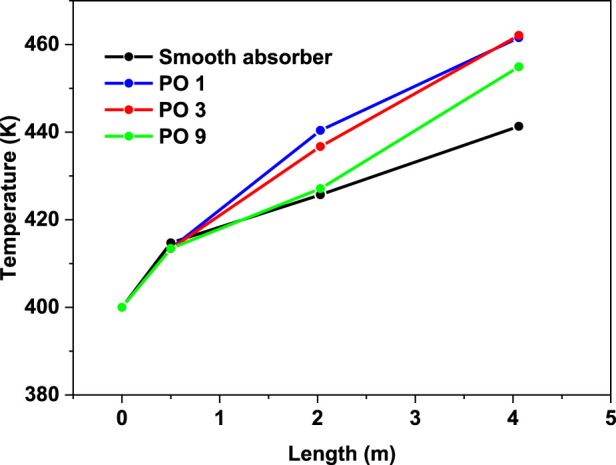
Variation in absorber tube’s average temperature and without perforated obstacles (Re = 81728, v = .65 m/s).

**FIGURE 12:**
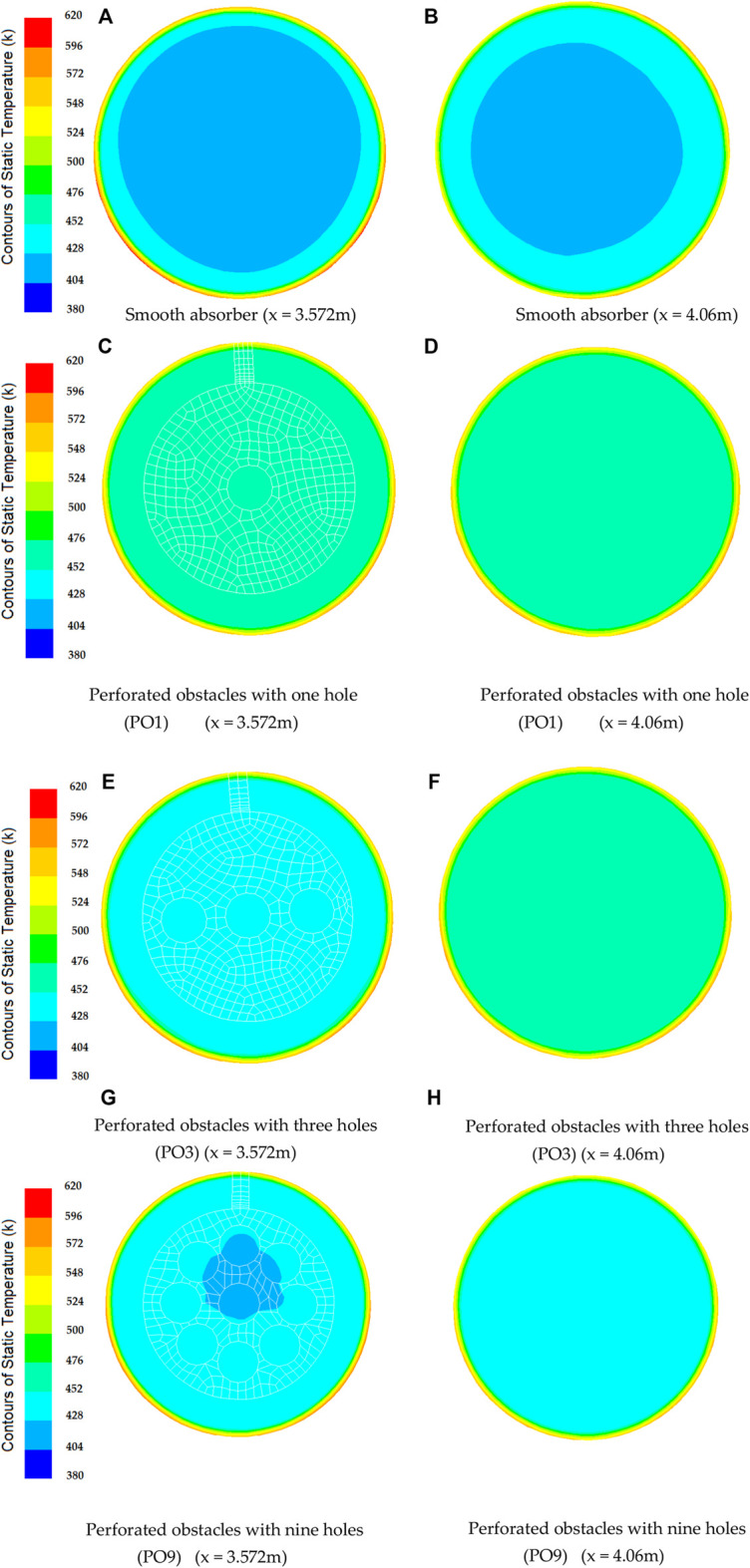
Temperature distribution of absorber tube of PTR at cross-sections with Re = 81728.

In contrast, perforated barriers with nine holes (PO9) have the smallest Nusselt number enhancement, which equals 54 percent. From Figure 10, it can be observed that the smooth case has the smallest friction factor of all the cases investigated in this study. The largest friction factor is obtained when perforated barriers with one hole (PO1) are used, followed by perforated barriers with three holes (PO3) and perforated barriers with nine holes (PO9) in the second and third cases, respectively. These higher values are caused by the whirling flow generated by the inserts that function as an obstruction. Figure 11 displays the heat transfer fluid average temperature distribution on sectional planes (y-axis and z-axis) along the entire length of the absorber tube with and without impediments. At the exit, the temperature reaches its peak. Higher temperatures (462.1 K) are achieved in the absorber tube with perforated barriers with three holes (PO3), followed by (461.56 K) for perforated barriers with one hole (PO1), and 454.92 K for perforated barriers with nine holes (PO9). Figure 12 shows the temperature distributions of the PTR absorber tube on two distinct cross-sections with Re = 81728 for various scenarios.

### Thermal performance analysis

To enhance heat transfer efficiency, it is required to assess both heat transfer and flow resistance concurrently. As a result, as stated below (Xiangtao et al., 2017), the performance evaluation criteria (PEC) are universal assessment tools that reflect a heat transfer unit’s overall performance. The thermal performance criterion was calculated as the ratio of the dimensionless Nusselt number to the dimensionless friction factor.

$$PEC=\frac{Nu/{Nu}_{0}}{{\left(f/f_{0\;}\right)}^{1/3}}$$ (19)

where (Nu0) and (Nu1) represent the smooth absorber case (f0).

**FIGURE 13:**
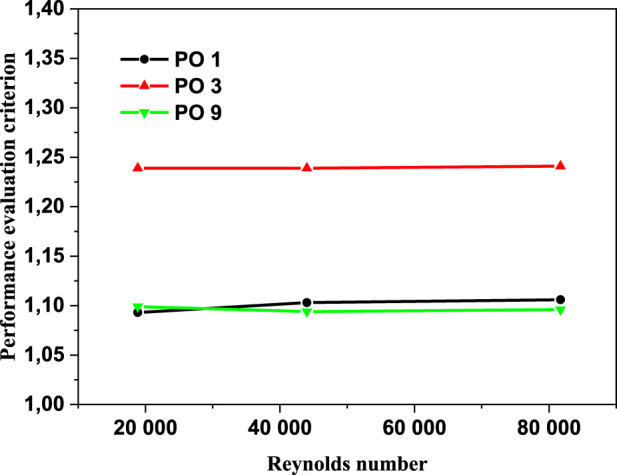
Performance evaluation criteria variation vs. Re number.

Figure 13 illustrates the fluctuation of performance evaluation criteria (PEC); when the PEC values exceed one, it indicates that the inserts have a favorable influence on heat transfer. It is notable that the perforated barriers give a heat transfer boost over the smooth tube. The perforated barriers with three holes had the greatest PEC value (PO3).

Table 5 includes the obtained increase in the Nusselt number (Nu) and in the performance evaluation criteria (PEC). Data has been estimated according to the results of these papers. Moreover, this table shows the method of every study. These studies more specifically, present lower performance evaluation criteria (PEC) compared to the present study. According to the final results, the use of perforated obstacles leads to 1.24% performance evaluation criteria (PEC) enhancement.

**TABLE 5:** Comparison with literature studies with inserts in the receiver of a parabolic trough solar collector and the present study.

Cas	Insert type	Increase (%) Nu	PEC	Method	Reference
**Present study**	Perforated obstacles	115	1,24	CFD	
**Gong Xiangtao**	Pin fin arrays	9	1.12	CFD	Mwesigye et al. (2016)
**Aggrey Mwesigye**	Twisted tape	58.8	1.02	CFD	Mwesigye et al. (2014)
**Aggrey Mwesigye**	Perforated plate	8–133.5	.44–1.05	CFD	

## Conclusion

The effect of utilizing varied perforated barriers on the thermal performance of parabolic through the solar receiver is computationally investigated in this work. The following observations could be drawn from this work.

• In comparison to the reference case (smooth absorber), the greatest increase in Nusselt number was 115%, and it was attained by the perforated obstacles with three holes (PO3), followed by 108% for the perforated obstacles with one hole (PO1), while the perforated obstacles with nine holes (PO9) achieved the minimum enhancement of 54%.
• Friction factor values decrease with the increase in the number of holes on obstacles. In the case of the tube without perforated obstacles, friction factor values are less than all the friction factor values with perforated obstacles inserts.
• The perforated barriers in the absorber tube increase the Nusselt number while decreasing the friction factor.
• The highest PEC value was obtained for the perforated obstacles with three holes (PO3).
• The temperature of the heat transfer fluid reaches its maximum value near the exit, while temperatures as high as 462.1 K are obtained in the absorber tube with perforated barriers with three holes (PO3).

## Data Availability

The original contributions presented in the study are included in the article/Supplementary Material, further inquiries can be directed to the corresponding author.
