# Corrigendum: Numerical study of perforated obstacles effects on the performance of solar parabolic trough collector

**DOI:** 10.3389/fchem.2023.1160174

**Published:** 2023-02-15

**Authors:** Tayeb Fahim, Samir Laouedj, Aissa Abderrahmane, Zied Driss, El Sayed Mohamed Tag-ElDin, Kamel Guedri, Obai Younis

**Affiliations:** ^1^ Materials and Reactive Systems Laboratory (LMSR), Djillali Liabes University, Sidi Bel Abbes, Algeria; ^2^ Laboratoire de Physique Quantique de la Matière et Modélisation Mathématique (LPQ3M), Université Mustapha Stambouli de Mascara, Mascara, Algeria; ^3^ Laboratory of Electromechanical Systems (LASEM), National School of Engineers of Sfax, University of Sfax, Sfax, Tunisia; ^4^ Center of Research, Faculty of Engineering, Future University in Egypt, New Cairo, Egypt; ^5^ Mechanical Engineering Department, College of Engineering and Islamic Architecture, Umm Al-Qura University, Makkah, Saudi Arabia; ^6^ Department of Mechanical Engineering, College of Engineering in Wadi Addwasir, Prince Sattam Bin Abdulaziz University, Al-kharj, Saudi Arabia

**Keywords:** nanofluid, parabolic trough solar collector, Nusselt number, perforated obstacles, numerical investigation

In the original article, there were errors in the **Funding** statement. The corrected statement appears below and replaces the **Acknowledgments**.

“The authors extend their appreciation to the Deputyship for Research & Innovation, Ministry of Education in Saudi Arabia for funding this research work through the project number: IFP22UQU4331317DSR174.”

The authors apologize for these errors and state that this does not change the scientific conclusions of the article in any way. The original article has been updated.

